# Respiratory distress in SARS-CoV-2 exposed uninfected neonates followed in the COVID Outcomes in Mother-Infant Pairs (COMP) Study

**DOI:** 10.1038/s41467-023-44549-5

**Published:** 2024-01-24

**Authors:** Olivia M. Man, Tamiris Azamor, Mary Catherine Cambou, Trevon L. Fuller, Tara Kerin, Sophia G. Paiola, Jessica S. Cranston, Thalia Mok, Rashmi Rao, Weiqiang Chen, Jae U. Jung, Viviana Fajardo Martinez, Suan-Sin Foo, Karin Nielsen-Saines

**Affiliations:** 1 David Geffen School of Medicine, University of California, Los Angeles, Los Angeles, CA 90095 USA; 2 Department of Cancer Biology, Infection Biology Program, Global Center for Pathogen Research and Human Health, Lerner Research Institute, Cleveland Clinic, Cleveland, OH 44195 USA; 3 Department of Medicine, Division of Infectious Diseases, David Geffen School of Medicine, University of California, Los Angeles, Los Angeles, CA 90095 USA; 4 Department of Pediatrics, Division of Infectious Diseases, David Geffen School of Medicine, University of California, Los Angeles, Los Angeles, CA 90095 USA; 5 Instituto Nacional de Infectologia Evandro Chagas, Fundação Oswaldo Cruz, Manguinhos, Rio de Janeiro, 21040-360 Brazil; 6 Department of Obstetrics and Gynecology, Division of Maternal-Fetal Medicine, David Geffen School of Medicine, University of California, Los Angeles, Los Angeles, CA 90095 USA; 7 Department of Pediatrics, Division of Neonatology, David Geffen School of Medicine, University of California, Los Angeles, Los Angeles, CA 90095 USA

**Keywords:** Epidemiology, Viral infection, SARS-CoV-2

## Abstract

Respiratory distress (RD) has been reported in SARS-CoV-2 exposed uninfected (SEU) term neonates. Prior studies suggest that prenatal exposure to Coronavirus Disease 19 (COVID-19) may activate an inflammatory cascade in the newborn airway. In this study, we examine the relationship between maternal COVID-19 vaccination and neonatal RD using a longitudinal cohort of mother-infant pairs in Los Angeles, CA. Two-hundred and twenty-one mothers with laboratory confirmed SARS-CoV-2 during pregnancy and 227 exposed fetuses are enrolled in our study. Maternal disease severity and neonatal RD variables were defined based on current accepted clinical criteria. To explore the multifactorial associations between maternal COVID-19 parameters and infant RD, we utilize a multivariable logistic regression model and a proteomic sub-analysis to propose a pathway for the development of RD following *in utero* exposure to SARS-CoV-2. Unusually high rates of RD are observed in SEU infants (17%). The odds ratio of RD is 3.06 (95% CI:1.08-10.21) in term neonates born to unvaccinated individuals versus those born to individuals vaccinated prior to maternal infection. Proteomic analysis reveals a robust inflammatory response associated with ciliary dysregulation and enhanced IgE production among SEU neonates with RD. Maternal vaccination against COVID-19 reduces the frequency of neonatal RD.

## Abstract (web summary)

Unusually high rates of respiratory distress were observed in infants following in utero exposure to SARS-CoV-2. Here the authors find, in a longitudinal cohort of infants born to persons infected with SARS-CoV-2 during pregnancy, that maternal vaccination is associated with a lower odds of infant respiratory distress.

## Introduction

SARS-CoV-2 infection during pregnancy has been associated with adverse maternal and neonatal outcomes, including increased risk of prematurity^1^, stillbirths^2^, and severe maternal morbidity and mortality^3^. While estimates of vertical transmission of SARS-CoV-2 from mother to child are low^1,4–6^, there is growing concern for long-term neonatal consequences^7^. Specifically, several authors have reported cases of infant respiratory distress (RD) among SARS-CoV-2-exposed uninfected (SEU) term neonates^8–10^.

Prior studies have attributed cases of RD among SEU neonates to maternal hypoxia and multiorgan failure, which increases the risk of premature delivery^11^, a well-studied risk factor for neonatal RD. However, this does not explain the increased cases of RD among infants born at term. Our prior analysis suggests that prenatal exposure to SARS-CoV-2 may activate an inflammatory cascade in the newborn airway leading to ciliary beat dysregulation of the airway epithelium^7^. Moreover, inflammasome associated proteins (IL-18, IL-1B, and CASP1) were found in SEU term infants with RD^7^. The exact mechanism of this pathway has yet to be elucidated.

Maternal immunization against COVID-19 has been shown to be highly protective against maternal mortality and morbidity^3^ and may prevent COVID-19 hospitalizations in infants under 6 months of age^12^. We have previously found an association between infant SARS-CoV-2 IgG levels at birth and maternal COVID-19 severity; however, maternal vaccination prior to delivery was the strongest predictor of detectable IgG at birth in their neonates^13^. The impact of maternal COVID-19 vaccination on neonatal RD following in utero exposure to COVID-19 remains unknown.

In this study, we explored the complex relationship between maternal SARS-CoV-2 infection during pregnancy and infant RD. To achieve this aim, we focused on the following objectives: (i) characterize the clinical features of RD among SEU infants; (ii) construct a multivariable statistical model to assess the effect of maternal vaccination on infant RD; and (iii) conduct a proteomic ingenuity pathway analysis to identify proteins differentially expressed in SEU infants and their biological functions. Our findings can help inform the mechanisms by which maternal SARS-CoV-2 infection during pregnancy may impact fetal development and neonatal outcomes. Moreover, our study highlights the importance of public health interventions and vaccination efforts that target pregnant individuals due to the potential for lasting effects on the health of both the mother and the infant.

## Results

### Participant demographics

**Fig. 1:**
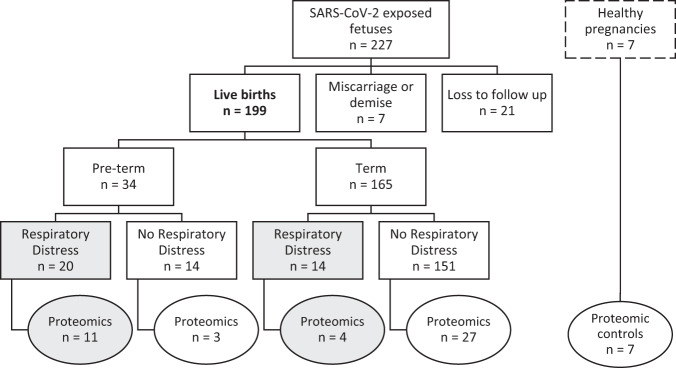
Flow diagram of study participants (*n* = 199) included in the main analysis and proteomic sub-analysis (*n* = 52). The 199 live births were included in our main statistical analysis; circles indicate the infants included in our proteomic sub analysis. Shaded boxes/circles highlight the neonates with respiratory distress. Healthy pregnancies have a dashed border to indicate that these participants were not included in our analysis.

During the study period, 221 pregnant persons with COVID-19 and 227 COVID-19 exposed fetuses were enrolled in our longitudinal cohort. One hundred and ninety nine infants were born between April 2020 and August 2022. Three percent of COVID-19 exposed fetuses (*n* = 7) resulted in a miscarriage, abortion, or fetal demise and 9% of COVID-19 exposed fetuses (*n* = 21) were lost to follow-up (Fig. 1). Most pregnant persons in our cohort self-identified as Black, Hispanic, or Latina (50%), followed by Asian, Mixed-Race, or Other (24%), and White (25%) (Table 1).

**Table 1:** Maternal study demographics (*n* = 221)

Variable	Overall
Maternal age (mean (s.d.))	32.68 (5.94)
Birth Outcome (%), *n* = 227^a^	
Live birth	199 (87.7)
Miscarriage, Abortion, or Demise	7 (3.1)
Loss to follow up (e.g., not delivered at UCLA)	21 (9.3)
Maternal Ethnicity (%)	
Asian, mixed race, or other	52 (24.0)
Black, Hispanic, or Latina	110 (50.7)
White	55 (25.3)
Maternal COVID severity^b^ (%)	
Asymptomatic, Mild, Moderate	192 (86.9)
Severe or Critical	29 (13.1)
Maternal vaccination prior to delivery (%)^c^	76 (37.3)
Maternal vaccination prior to infection (%)^c^	70 (33.8)
Neonatal Respiratory Distress (%), *n* = 227^a^	34 (17.0)

^a^Two hundred and twenty-one mothers were enrolled in our study, corresponding to 227 SARS-CoV-2 exposed fetuses due to multiple gestations.

^b^COVID severity index was determined based on the NIH classification system^14^, as done by Thompson et al.^4^. Briefly, critical illness describes patients with respiratory failure or signs of multiple organ failure, severe illness was defined as patients with desaturations requiring supplemental oxygen, moderate illness included patients with dyspnea or who were admitted to an inpatient unit for COVID, mild illness describes symptomatic patients not meeting criteria for more severe disease, and asymptomatic individuals showed no symptoms.

^c^Individuals were eligible to be included in both categories depending on the sequence of infection, vaccination, and delivery.

**Fig. 2:**
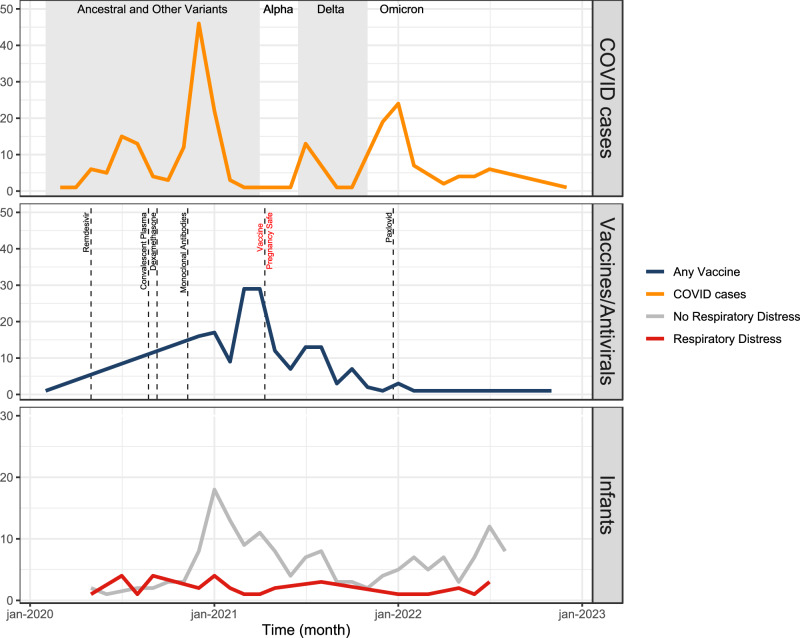
Maternal COVID cases (*n* = 221), maternal immunizations (*n* = 70), and cases of infant respiratory distress (*n* = 34) overtime. **A** COVID Cases: The gray shaded regions represent when different variants were circulating in California. Ancesteral and other variants circulated from 1 Feburary 2020 to 1 April 2021; the Alpha variant circulated from 1 April 2021 to 15 June 2021; the Delta variant circulated from 15 June 2021 to 15 December 2021; and the Omicron variant circulated from 15 December 2021 onward. **B** Immunizations: Dashed vertical lines signify key changes in treatment availability. On 1 May 2020 the FDA issued an emergency use authorization (EUA) for Remdesivir, on 23 August 2020 the FDA issued an EUA for convalescent plasma, on 3 September 2020 the Journal of the American Medical Association and the WHO recommended use of dexamethasone, on 9 November 2020 the FDA issued an EUA for use of monoclonal antibodies, on 11 August 2021 the CDC confirmed that vaccines were safe during pregnancy, and on 22 December 21 the FDA authorized Paxlovid use^33^. **C** Infant respiratory distress over time with red representing infants born with respiratory distress and gray representing infants born without respiratory distress.

Thirteen percent of pregnancies (*n* = 29) met the National Institutes of Health (NIH) criteria^14^ for severe or critical COVID-19 (Table 1). One hundred and fifty-one mothers (68%) were unvaccinated with severe or critical disease present in 16% (*n* = 23); whereas only 4% of vaccinated mothers (*n* = 3) had severe or critical disease (Table 1). The greatest number of COVID-19 cases among mothers in this cohort was in winter 2020, when the ancestral variants were circulating (Fig. 2). This was followed by a smaller peak around the emergence of the delta variant and a larger peak coinciding with the emergence of omicron (Fig. 2). Most pregnant patients received a COVID-19 vaccine prior to the circulation of the alpha variant (Fig. 2). There was a significant difference among maternal vaccination status and predominant circulating variant at the time of infection (*p* < 0.001, Supplementary Table 1).

None of the infants in our study tested positive for SARS-CoV-2 at birth (Table 1) and 17% (*n* = 34) were diagnosed with RD (Table 2). Incidence of RD remained stable over time and did not appear to change with the emergence of new variants and availability of maternal vaccinations (Fig. 2). Of the infants with RD, 38% (*n* = 14) were full term and 68% (*n* = 23) had a low birth weight (<2500 g) (Table 2). There was not a significant difference among infants with and without RD born to pregnant patients with preexisting maternal comorbidities (Table 2). However, a much higher percentage of infants with RD were born to pregnant persons that developed a severe hypertensive disorder of pregnancy (HDP) compared to infants without RD (Table 2, Supplementary Fig. 1). In other words, neonatal RD, prematurity, and severe HDP were not independent by chi-squared testing (*p* < 0.001). Only 15% of infants with RD were born to mothers vaccinated prior to SARS-CoV-2 infection, as opposed to 41% of infants without RD (*p* = 0.012, Table 2). The prevalence of severe or critical COVID-19 in mothers was significantly higher among infants with RD compared to those without RD (21% vs 6%, respectively; *p* = 0.009) (Table 2).

**Table 2:** Maternal and infant demographics stratified by neonatal respiratory distress (RD)

Variables	No RD (*n* = 165)	RD (*n* = 34)	*P* value^*^
Infant outcomes			
Male Sex (%)	80 (48.5)	18 (52.9)	0.776
Vaginal Delivery (%)	112(67.9)	8 (23.5)	<0.001
Preterm Delivery <37 wks	14 (8.5)	20 (58.8)	<0.001
Low birth weight (<2500 g)	9 (5.5)	23 (67.6)	<0.001
Maternal Predictors			
Age (mean yrs (s.d.))	32.62 (6.02)	33.41 (5.82)	0.483
Ethnicity			0.479
Asian, mixed race, or other	42 (25.5)	5 (15.6)	
Black, Hispanic, or Latino	80 (48.5)	17 (53.1)	
White	43 (26.1)	10 (31.2)	
Pulmonary Hypertension	1 (0.6)	0 (0.0)	1.000
Autoimmune disorder	15 (9.1)	3 (8.8)	1.000
HIV positive	2 (1.2)	0 (0.0)	1.000
Obesity pre-pregnancy	42 (25.5)	13 (38.2)	0.191
Diabetes	7 (4.2)	2 (5.9)	1.000
Congenital Heart Disease	8 (4.8)	0 (0.0)	0.406
Asthma	20 (12.2)	3 (8.8)	0.800
Substance Use	8 (4.9)	0 (0.0)	0.414
Pregnancy Complications			
Chronic or Gestational Hypertension	43 (26.1)	17 (50.0)	0.010
Preeclampsia or HELLP	18 (10.9)	13 (38.2)	<0.001
Gestational Diabetes	27 (16.4)	7 (20.6)	0.730
Chorioamnionitis	15 (9.1)	0 (0.0)	0.141
Postpartum Hemorrhage	11 (6.7)	9 (26.5)	0.001
COVID Predictors			
Vaccination prior to infection	63 (40.9)	5 (15.6)	0.012
Vaccination prior to delivery	69 (41.8)	6 (18.8)	0.024
Trimester of SARS-CoV-2 infection			1.000
First or second	79 (47.9)	16 (47.1)	
Third	86 (52.1)	18 (52.9)	
COVID severity			0.009
Asymptomatic, Mild, or Moderate	156 (94.5)	27 (79.4)	
Severe or Critical	9 (5.5)	7 (20.6)	
COVID symptoms			
Fever	40 (25.2)	11 (39.3)	0.188
Cough, Rhinorrhea, Congestion	130 (81.8)	16 (59.3)	0.017
Dyspnea	27 (17.0)	9 (31.0)	0.130
Nausea/Vomiting, Abdominal Pain, Diarrhea	25 (15.7)	0 (0.0)	0.056
Anosmia or Dysgeusia	37 (23.3)	6 (22.2)	1.000
Treatment			
Remdesivir	12 (7.3)	7 (21.2)	0.031
Dexamethasone	6 (3.6)	7 (21.2)	0.001
Convalescent Plasma	0 (0.0)	4 (12.2)	<0.001
Monoclonal Antibodies	13 (7.9)	2 (5.9)	0.964
Paxlovid	2 (1.2)	1 (3.0)	1.000
Variant			0.757
Ancestral and Others	88 (53.3)	19 (63.3)	
Alpha	1 (0.6)	0 (0.0)	
Delta	23 (13.9)	3 (10.0)	
Omicron	53 (32.1)	8 (26.7)	

*p values were calculated based on one-sided Fisher’s exact test. We did not adjust for multiple comparisons because this portion of our analysis was exploratory.

### Clinical features of respiratory distress among SEU infants (Objective I)

Of the 34 infants in our study with RD, the most common diagnosis upon discharge from the neonatal intensive care unit was Respiratory Distress Syndrome (47%), followed by Transient Tachypnea of the Newborn (16%), and Other Infection (16%) (Table 3). There were no neonatal deaths. While many of the infants were considered early preterm at less than 34-weeks gestation (*n* = 15), the majority of infants were late preterm (*n* = 6) or term (n = 13) deliveries (Table 3). The average time to resolution of RD was 24.38 days (s.d. = 25.02) with a median time of 15 days (IQR: 8.3, 29.5). The time to resolution varied by gestational age (early preterm infants med = 30 (IQR: 13.5, 54.5); late preterm infants med = 14.5 (IQR: 13, 18.3); term infants med = 7 (IQR: 2, 15)). Physical exam findings were not specific and often included descriptors such as subcostal or intercostal retractions (27%), abnormal respirations (14%), or grunting or nasal flaring (14%) (Table 3). The most common chest X-ray findings involved various descriptors of opacification (i.e., interstitial opacities, ground glass opacities; 20%) (Table 3). However, 8% of chest X-rays were described as normal (Table 3).

**Table 3:** Descriptors of infants with respiratory distress (*n* = 34)

Variable	Total (%)
Gestational age	
Preterm	15 (44.1)
Late Preterm	6 (17.6)
Term	13 (38.2)
Diagnosis at NICU discharge^a^	
RDS	21 (46.7)
TTN	7 (15.6)
Infection (i.e., sepsis, pneumonia)	7 (15.6)
Apnea of prematurity	3 (6.7)
Other	7 (15.6)
Reported Physical Exam Findings^a^	
Subcostal/intercostal retractions	20 (27.0)
Abnormal respirations (i.e., tachypneic, apneic)	10 (13.5)
Grunting or nasal flaring	10 (13.5)
Poor respiratory effort	8 (10.8)
Skin discoloration (i.e., cyanotic, dusky)	8 (10.8)
Desaturating on a monitor	8 (10.8)
Abnormal breath sounds (i.e., coarse, crackles)	5 (6.8)
Another descriptor	5 (6.8)
Chest X-Ray findings^a^	
Opacities (i.e., interstitial, ground glass, etc.)	20 (40.0)
Low lung volumes	10 (20.0)
Excess fluid (i.e., interstitial edema, fluid, vascular congestion)	9 (18.0)
Other (i.e., atelectasis, etc.)	7 (14.0)
Normal CXR	4 (8.0)

^a^Multiple findings/categories were often reported for diagnosis at NICU discharge, physical exam findings, and chest X-ray findings; therefore, the totals of these categories may exceed the total number of infants with respiratory distress.

### Maternal vaccination and infant respiratory distress (Objective II)

In the unadjusted logistic regression models, neonatal RD was associated with maternal disease severity (OR: 4.50, 95%CI: 1.49–13.10), prematurity (OR: 15.41, 95%CI: 6.55–38.07), and absence of maternal COVID-19 immunization (OR: 3.74, 95%CI: 1.47–11.49) (Supplementary Fig. 3). We did not observe an association between RD and trimester of maternal COVID-19 infection pregnancy (OR: 1.03, 95%CI: 0.49–2.18) nor maternal race/ethnicity (OR: 0.91, 95%CI: 0.39–2.23) (Supplementary Fig 3).

**Fig. 3:**
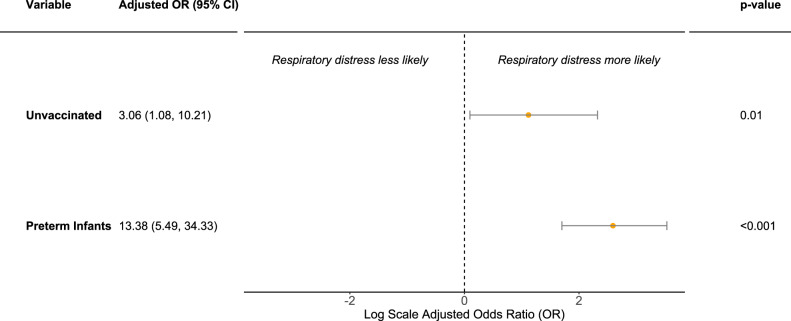
Multivariable logistic regression analysis of respiratory distress in neonates born to mothers infected with COVID during pregnancy (*n* = 199). OR odds ratio, CI Confidence interval. Data are presented as the OR ± 95% CI; *p* values are calculated based on one-sided Wald’s test. Multivariable logistic regression model included maternal vaccination status and infant prematurity as predictors for respiratory distress. Unvaccinated refers to mothers who did not receive a COVID-19 vaccine compared to vaccinated mothers (reference group) who received at least 1 mRNA COVID-19 vaccine prior to infection. Preterm infants refer to neonates born at less than 37 weeks gestation compared to term infants (reference group) born at greater than or equal to 37 weeks gestation.

In the multivariable logistic regression model, holding prematurity constant, the odds ratio of RD in neonates born to unvaccinated women was 3.06 (95% CI:1.08-10.21) (Fig. 3). When pregnant patients received at least one messenger RNA (mRNA) vaccine dose prior to infection, the OR of neonatal RD was 0.33 (95%CI: 0.10—0.96), a 67% decline. An alternate multivariable logistic regression model showed that the relationship between maternal disease severity and neonatal RD, holding prematurity constant, was in the expected direction but was not significant (OR: 2.10, 95%CI: 0.55, 7.62) (Supplementary Fig. 4).

### Proteomic pathway analysis (Objective III)

We analyzed 52 infants born during the first year of the pandemic (April 2020 to March 2021) using proteomic techniques. A subset of 45 SEU infants (23% of 199 included in our statistical analysis) were matched to 7 control infants born from healthy unexposed pregnancies. Previously, among SEU infants with RD born at all gestational ages, we observed elevated levels of Interleukin 18 (IL-18), Interleukin 1 beta (IL-1B), and Caspase 1 (CASP1)—indicating upregulated NACHT, LRR, and PYD domains-containing protein 3 (NLRP3) inflammasome mediated pathway^7^. Moreover, we identified the following 5 unique cytokines that were elevated among SEU term infants with RD: iduronidase (IDUA), Triggering Receptor Expressed on Myeloid Cells 2 (TREM2), Ghrelin and Obestatin Prepropeptide (GHRL), anterior gradient 3, protein disulfide isomerase family member, and procollagen C-endopeptidase enhancer (PCOLCE)^7^. Further evaluation with a complete Ingenuity Pathway Analysis (IPA) was not possible given the limited number of unique cytokines identified for this group.

**Fig. 4:**
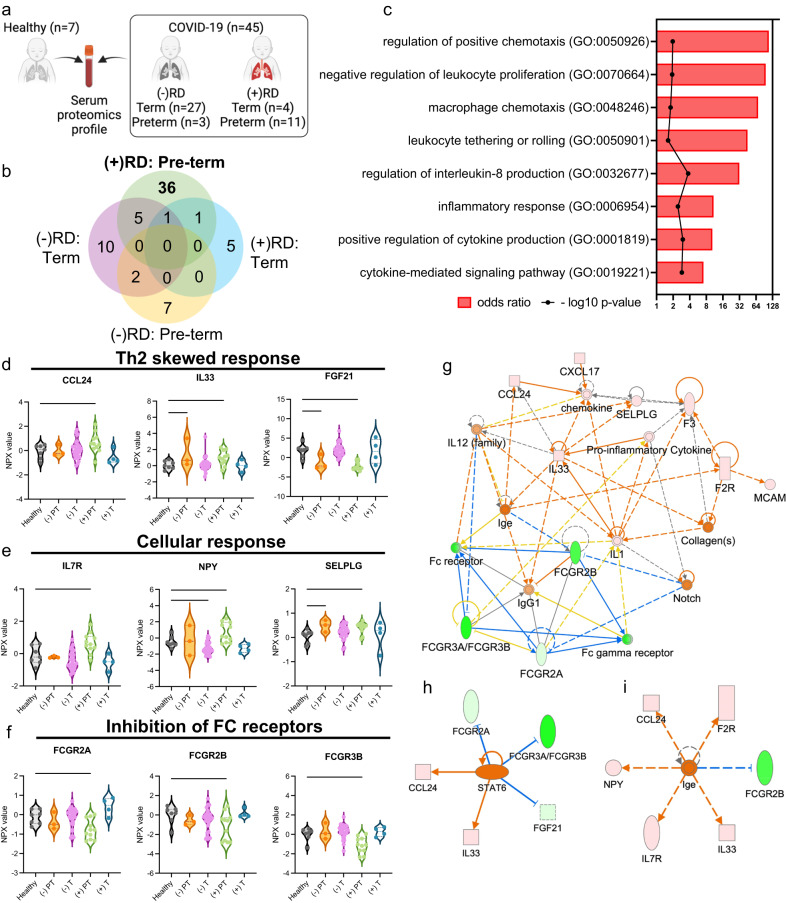
Proteomic analysis in a subset of SARS-CoV-2 exposed uninfected infants (*n* = 45) compared to control infants born to healthy mothers (*n* = 7). a. Schematic representation of serum proteome multiplexing of COVID-19-affected (COVID-19) infants. Infant blood specimens were collected from infants within the first 2 days of life born to healthy mothers (*n* = 7) or to SARS-CoV-2-infected mothers (*n* = 45). COVID-19-exposed infants were clustered according to pregnancy duration and the absence (-) (preterm *n* = 3, and term *n* = 27) or presence (+) (preterm *n* = 11, and term *n* = 4) of respiratory distress (RD). Created with BioRender.com. **b** Quantitative comparison of cytokines significantly altered (*p* < 0.05; −2 < FC > 2) in COVID-19 infants with or without respiratory distress, (−) or (+) RD, respectively, in contrast to cytokines observed in pre-term and term pregnancy in both groups. Quantitative analysis in all groups relative to healthy controls, *p* values were determined based on two-tailed Mann Whitney *U* Test considering fold-change ≥2 and FDR-adjusted p-value < 0.05. c. Enricher, Gene ontology biological pathways upregulated exclusive for COVID-19-exposed infants (+) RD pre-term compared with healthy controls; *p* values obtained from a one-way ANOVA with uncorrected Fisher’s test. Proteins upregulated and downregulated in infants from COVID-19 pregnancy (+) RD pre-term, related with (**d**) Th2 skewed response, (**e**) activation of cellular response and (**f**) inhibition of FC receptors. Data are presented as means ± SEMs, using 1-way one-way ANOVA with uncorrected Fisher’s test. ^*∗*^*p* < 0.05, ^∗∗^*p* < 0.01, ***p < 0.005, ****p < 0.001; please see text for exact *p* values. **g** IPA predicted functional networks exclusive for COVID-19 pregnancy (+) RD pre-term compared with healthy controls. **h** Proteins differentially expressed (PDEs) associated with Activation of Signal Transducer and Activator of Transcription 6 (STAT6) pathways among SARS-CoV-2 exposed uninfected preterm neonates. **i** PDEs associated with higher IgE production among SARS-CoV-2 exposed uninfected preterm neonates.

Here, we observed 36 proteins differentially expressed (PDE) among SEU preterm infants with RD (Fig. 4a, b). Our enrichment analysis demonstrated that preterm infants with RD had significantly upregulated biological processes related to inflammation (OR = 10.87, *p* = 0.0036), chemotactic responses (OR = 109.70, *p* = 0.0108), and cellular responses, such as Interleukin 8 (IL8) production (OR = 31.88, *p* = 0.0002; Fig. 4c). Compared with healthy infants, SEU preterm infants with RD had elevated levels of Th2-associated cytokines C-C Motif Chemokine Ligand 24 (CCL24; *p* = 0.0461) and Interleukin 33 (IL33; *p* = 0.0458), together with low levels of the Th2-inhibitor Fibroblast Growth Factor 21 (FGF21; *p* < 0.0001) (Fig. 4d). This group also had higher levels of cytokines related to cellular proliferation and adhesion when compared to the controls, including Interleukin 7 Receptor (IL7R; *p* = 0.0076), Neuropeptide Y (NPY; *p* = 0.009), and Selectin P Ligand (SELPLG; *p* = 0.0188) (Fig. 4e). Additionally, we observed downregulation of FC gamma receptor 2A (FCGR2A; *p* = 0.0087), 2B (FCGR2B; *p* = 0.031), and 3B (FCGR3B; *p* = 0.0026) among SEU preterm infants with RD compared to control infants (Fig. 4f). The analysis of functional networks composed of the 36 PDE also suggests that SEU preterm infants with RD present mainly a Th2-skewed response reinforced by the FC gamma receptor inhibition (Fig. 4g), and activation of Signal Transducer and Activator of Transcription 6 (STAT6) pathway (Fig. 4h)^15^. This anti-inflammatory response could move towards hyperimmune responses, due to PDEs association with higher Immunoglobulin E (IgE) production (Fig. 4i).

## Discussion

We analyzed a cohort of individuals infected with COVID-19 during pregnancy and their neonates to better understand the effects of in utero COVID-19 exposure on neonatal RD. We observed high rates of RD in SEU infants. The prevalence of severe or critical COVID-19 was higher among unvaccinated mothers. The odds of neonatal RD were significantly reduced among term infants if mothers received at least one mRNA COVID-19 vaccine prior to infection (Fig. 3). Our proteomic sub-analysis revealed a robust inflammatory response among SEU infants and the main pathways involved appear to be associated with ciliary function, macrophage activation, and enhanced IgE production. Our results demonstrate that maternal vaccination against COVID-19 not only protects against maternal disease severity, but also reduces the likelihood of neonatal RD.

While many SEU infants with RD were early preterm deliveries (less than 34-weeks gestation), the majority were either late preterm or term neonates (Table 3). In the unexposed population, the overall incidence of RD has an estimated range of 5.2–6.4% and a well-established inverse relationship with gestational age^16^. Premature infants are at a greater risk for RD due to underdeveloped lung anatomy and insufficient surfactant production prior to 35-weeks of gestation^17^. Not only do our results show higher rates of RD in SEU infants when compared to the general population but we observed more cases of RD at later gestational ages than anticipated, when neonates should presumably have more mature lung anatomy.

Immunologic evaluation of SEU neonates with RD identified a heightened inflammatory response (Fig. 4a-i). Previously, we found evidence of an upregulated NLRP3 inflammasome-mediated pathway in SEU neonates with RD born at all gestational ages^7^. Among preterm SEU neonates with RD, our IPA showed Th2-skewed immune activation. This was reinforced through inhibition of FC gamma receptors and upregulation of the STAT6 pathway, which includes IL-33. Th2-skewed responses have been previously identified in children with COVID-19 when compared to adults who tend to favor a Th1-skewed response^18^. During an infection, Th2-skewed responses have been associated with lower levels of pro-inflammatory cytokines and less severe COVID-19 presentations^18,19^. In our cohort of SEU neonates, a Th2 response may be more indicative of robust IgE-mediated inflammation (Fig. 4i). This may occur as Th2 cells stimulate production of IgE antibodies, which sensitize various cells through binding of FC receptors^20^. This enhanced IgE production may shift the neonatal response to a hyperinflammatory state with long-term implications that include the development of various allergic processes^15^.

Moreover, SARS-CoV-2 antibody-virus immune complexes bind to the FC receptors on alveolar macrophages leading to the release of pro-inflammatory factors, such as IL-8^21^. Inhibition of the FC receptors (Fig. 4f) may lead to increased antibody levels and a prolonged immune response. Prolonged Th2 responses have been previously associated with cytotoxic damage and eventual tissue fibrosis^22^. Th2 immunity may be related to elevated levels of IL-33, which is thought to induce further pulmonary fibrosis^23^. We also observed elevated levels of cytokines related to cellular proliferation and adhesion (Fig. 4e), which may contribute to the hyperinflammatory and fibrosis picture. Finally, we detected upregulated IL-8 production (Fig. 4c), which has been previously identified in COVID-19 patients^19,24,25^ and implicated in the pathogenesis of adult Acute Respiratory Distress Syndrome (ARDS)^26^. Thus, these neonates may not only be at risk of developing a hyperinflammatory state at birth, but may have worsening pulmonary function over time due to altered respiratory anatomy.

Among SEU-term neonates with RD, we have previously identified elevated TREM2 levels responsible for promoting macrophage survival and viral induced lung pathogenesis; decreased AGR3 levels that are essential in regulating the airway ciliary beat frequency; and upregulated IDUA levels found to degrade glycosaminoglycans found in the extracellular matrix of the airway^7^. Moreover, we found that pregnancies with COVID-19 developed more pronounced inflammatory responses with activation of the IFNL1/IFNLR1 axis, which may trigger perinatal immune activation^7^. Other authors have found SARS-CoV-2 RNA in the placenta of infected mothers with elevated immune cells and inflammation^27^. Importantly, our prior analyses show that these maternal cytokines and chemokines do not appear to cross the placenta^7^. In other words, the presence of inflammatory proteins in infants cannot be explained by the transfer of inflammatory products across the placenta from their mothers and this is likely the infant’s own inflammatory response to maternal inflammation. This sustained inflammation and dysregulation of signaling pathways may lead to asynchronous respiratory function with unknown long-term sequalae.

The relationship between COVID-19 vaccination and the reduction of disease severity has been well established^28^. Pregnant individuals with severe or critical COVID-19 may require mechanical ventilation or Extracorporeal Membrane Oxygenation (ECMO)^4,29^. Maternal COVID-19 severity has been previously associated with poor infant outcomes^30^ although detectable SARS CoV-2 IgG levels are often present in neonates at birth^13^. While maternal COVID-19 severity was initially significant in our univariate analysis, this effect was attenuated once maternal vaccination was incorporated in the model. This finding is not unexpected, given the relationship between vaccination and maternal COVID-19 severity. Here our findings highlight that maternal vaccination may also decrease infant morbidity in SEU neonates.

Other authors have found an association between full maternal vaccination (completion of a two-dose vaccine series) and improved infant outcomes^12^. Only three infants in our cohort with RD were born to mothers who had completed the two-dose series, limiting our ability to statistically assess the effect of full vaccination versus partial vaccination on infant RD. Nevertheless, the small number of infants with RD born to women who completed the two-dose series highlights the potential benefit of additional maternal vaccine doses in reducing poor infant outcomes. Moreover, our results suggest that even maternal partial vaccination (greater than or equal to 1 mRNA vaccine) is still protective against neonatal RD. This is especially relevant in areas with limited access to vaccinations or large numbers of patients lost to follow up between vaccine doses.

Emergence of the delta variant has been previously associated with more severe outcomes during pregnancy, including 3.3 times the increased risk of death when compared to other variants^31,32^. For infants, maternal immunization has been previously shown to have the greatest benefit in reduction of infant hospitalizations during circulation of the Delta variant^12^. We did not observe a relationship between SARS-CoV-2 variants and maternal severity or infant RD. In part, our findings may be explained by the relatively large number of maternal COVID-19 cases during the circulation of the ancestral variants compared to the number of cases during the circulation of alpha, delta, and omicron strains (Fig. 2). Moreover, while a significant proportion of pregnant persons who gave birth to infants with RD were unvaccinated prior to infection (Table 1), a large proportion of pregnancies in our cohort overall received at least one vaccination prior to the emergence of the new variants (Fig. 2). For example, we observed very few cases of infected pregnant patients during the circulation of the alpha variant. This may be due to the large peak in vaccinations prior to the emergence of the alpha strain and the CDC declaration that the mRNA COVID-19 vaccines are safe during pregnancy^33^ (Fig. 2). It is likely that early vaccination provides some degree of protection against the newly emerging strains for mothers and neonates. Nevertheless, it is somewhat puzzling that the frequency of infant RD following maternal SARS-CoV-2 infection remained relatively constant throughout the study period, and did not necessarily diminish with the appearance of less virulent variants of concern, likely secondary to improved maternal immunity. It is possible that as the virus continues to evolve and transmission becomes more endemic, more individuals will have been exposed prior to pregnancy, which may be what we start to observe towards the end of our study period. This may reduce the severity of disease among pregnant persons, but not necessarily meaningfully reduce the inflammatory cascade that develops in their neonates. Additionally, this inflammatory cascade may be augmented by certain conditions of pregnancy, such as hypertensive disorders.

A large percentage of infants with RD were born to pregnant persons with a HDP compared to infants without RD (Table 2). We did not include these factors in our statistical model due to data sparsity and concern for multicollinearity between severe HDP and prematurity (Supplementary Fig. 1), as treatment for severe preeclampsia and HELLP involve urgent delivery. The relationship between severe HDP and severe COVID-19 is further complicated due to their similar clinical presentations^34,35^. Moreover, mounting evidence suggests that SARS-CoV-2 infection in pregnancy is an independent risk factor for the subsequent development of preeclampsia^36,37^. In our study, the distribution of infants with RD born to mothers without HDP was relatively even across all gestational ages (Supplementary Fig. 1). On the other hand, in mothers diagnosed with preeclampsia/HELLP, the majority of their infants with RD were also premature (Supplementary Fig. 1). While our study was not powered sufficiently to determine the effect of severe HDP on SEU infants with RD adjusting for prematurity (Supplementary Fig. 1), our preliminary analysis suggests that these hypertensive disorders may act as an intermediate or modify the effect of the relationship between in utero COVID-19 exposure and RD (Supplementary Fig. 2).

There are several limitations to our study. The majority of pregnant individuals in our cohort were enrolled from a tertiary and quaternary medical center in Los Angeles, California. Several mother-infant pairs were transferred from community hospitals in the county due to severity of their symptoms. Therefore, our study population may be skewed towards more severe presentations of COVID-19 compared to the general pregnant population. On the other hand, due to the high level of specialized services and medical equipment available, our study may also have higher rates of survival when compared to other regions. We did not collect data on the effects of natural SARS-CoV-2 infections prior to pregnancy or of vaccinations after natural infections. These two scenarios may impact the severity of maternal disease and the ultimate effect on fetal development. While natural infection prior to pregnancy likely does provide some benefit for SEU neonates, prior analyses have found the highest levels of infant IgG among those who received mRNA vaccines during pregnancy^13,38–42^. Additionally, the incidence of neonatal RD in the general population is low. While our findings suggest that the magnitude of the effect of in utero COVID-19 exposure on neonatal RD is high; the absolute number of cases remains small and limits the power of our study. Given our small sample size, our results should be interpreted with caution. Our proteomic analysis cannot definitively exclude all other causes of inflammation at the maternal-fetal interface leading to similar or overlapping inflammatory patterns. It is certainly possible that other disease states may share components of this inflammatory footprint and further research is needed to evaluate these differences. Finally, the effect of maternal COVID-19 treatments and the possibility of attenuating the risk of RD in the infant is difficult to interpret due to the following: treatments were available at different times during the pandemic; treatments are recommended for different levels of COVID-19 severity; and treatment recommendations change depending on the predominant circulating variant. Nevertheless, relatively few pregnant persons received these treatments in our cohort (Table 2). It is possible that these treatments may impact neonatal RD, but our study is not adequately powered to analyze this relationship.

In conclusion, we observed unusually high rates of RD among SEU infants. Maternal vaccination against COVID-19 reduced maternal disease severity and the frequency of neonatal RD. Pregnant persons should be encouraged to receive mRNA COVID-19 vaccines, regardless of history of prior COVID-19 infection. More research is needed to understand the impact of maternal COVID-19 vaccination on long term infant health and development, including chronic pulmonary conditions.

## Methods

### Study site, population, and study design

The COVID-19 Outcomes in Mother-Infant Pairs (COMP) study is a longitudinal cohort study of pregnant patients who had SARS CoV-2 during gestation and their infants^7,13^. Pregnant individuals, ≥16 years old or older, with confirmed SARS-CoV-2 infection by nasopharyngeal reverse transcription polymerase chain reaction (RT-PCR), antigen (Ag) or serology during gestation were eligible for enrollment, regardless of preexisting conditions. Participants were primarily recruited by the Department of Obstetrics at the University of California, Los Angeles (UCLA) from 15 April 2020 to 31 August 2022. Beginning in April 2020, all women admitted to UCLA labor and delivery were screened for SARS-CoV-2 by nasopharyngeal swab. Two-hundred and twenty-one pregnant individuals, aged 16 to 56 years old, and 227 SARS-CoV-2 exposed fetuses were enrolled in our study. This resulted in 199 live births following in utero exposure to COVID-19. Maternal-infant pairs were followed longitudinally until the infants reached 6 months of age. The UCLA Medical Center comprises of multiple teaching hospitals, including tertiary and quaternary referral centers, and services the Los Angeles region in Southern California.

Informed consent for participation was obtained for all participants prior to enrollment. If a participant was incapable to provide consent (i.e., due to an acute hospitalization or intubated), consent was provided by a surrogate decision maker and the participant was re-consented once they regained capacity. Information was obtained directly from participants using RedCap survey software and from clinical chart review using EPIC. Our study was approved by the UCLA Internal Review Board (IRB).

### Sampling, variables, and definitions

Our primary outcome was neonatal RD, which was defined as infants with at least two of the following: respiratory rate of 60 breaths per minute, retractions, nasal flaring, or central cyanosis. Infants were considered premature if they were born at a gestational age less than 37 weeks. Maternal COVID-19 severity was determined by NIH classification^14^. Briefly, maternal critical illness describes patients with respiratory failure (requiring mechanical ventilation) or signs of multiple organ failure; severe illness was defined as patients with oxygen saturation (SpO_2_) < 94% on room air, a ratio of arterial partial pressure of oxygen to fraction of inspired oxygen (PaO2/FiO_2_) < 300 mmHg, a respiratory rate >30 breaths/min, or lung infiltrates >50%; moderate illness was defined as individuals with evidence of lower respiratory disease on clinical assessment or imaging with a SpO_2_ ≥ 94% but did not require supplemental oxygen; mild illness describes symptomatic patients without shortness of breath, dyspnea or abnormal chest imaging; and asymptomatic individuals showed no symptoms^14^. Women were considered vaccinated if they received at least one dose of an mRNA COVID-19 vaccine prior to infection. Women who received the Janssen/Johnson & Johnson COVID-19 during pregnancy were not included (*n* = 1).

Maternal race and ethnicity were operationalized into three categories (Black, Hispanic, and Latina; Asian, Mixed-Race, and Other; or White) based on self-reported racial identity. We acknowledge that race is a social construct and our categorizations may not adequately reflect an individual’s identity. However, we included race in our univariate analysis given the history of systemic racism that has contributed to poor maternal outcomes among black women in the United States^43^. None of the mothers in our study self-identified as non-binary or transgender, therefore we have used gendered language in our text to refer to pregnant persons.

### Statistical analysis and model construction

We compared the demographics of infants born with and without RD using one-way t-tests. We considered variables related to infant characteristics (sex, delivery method, prematurity, low birth weight), maternal predictors (maternal age, ethnicity, preexisting medical conditions), pregnancy complications (e.g., preeclampsia, gestational hypertension, chorioamnionitis, etc.), and COVID-19 predictors (maternal vaccination, trimester of infection, severity, symptoms, treatment, viral variant). The Fisher exact statistical test was used to obtain p-values. We did not adjust for multiple comparisons in our bivariate analyses because it was exploratory. Next, we conducted logistic regression analyses on neonatal RD, prioritizing variables that were significant in the previous t-tests and based on clinical suspicion of intermediate variables and effect modifiers. The following variables were used as predictors in our univariate analysis: maternal ethnicity, trimester of infection, COVID-19 severity, maternal vaccination status, and binary prematurity. We selected variables to include in our final model using a backwards selection and WALDs test. As recommended by ref. l^44^, we initially included all variables in a model and eliminated each variable above the p-value threshold of 0.25. This process was repeated until the remaining variables had *p* values below the threshold. We considered potential collinearity or lack of independence among predictor variables using chi-squared tests for independence. Similarly, our final multivariable model did not include maternal COVID-19 severity—despite evidence of significance in univariate regression models—because of the known association between COVID-19 severity and vaccination status. Including both COVID-19 severity and vaccination status would have likely attenuated the strength of our findings. In order to evaluate whether prematurity mediates the effect of maternal COVID-19 on neonatal respiratory distress, we utilized the Valeri and Vanderweele mediation analysis^45^. Approximately 18% of the risk of neonatal respiratory distress is mediated by prematurity, although not statistically significant (*p* = 0.3). Our final model for neonatal RD included binary maternal vaccination status and infant prematurity as predictors. Finally, we conducted a chart and imaging review to better characterize features of infant RD born to women infected with COVID-19 during pregnancy.

We performed a post-hoc power analysis in which we calculated the power achieved in our study based upon the false positive rate, the effect size of maternal COVID-19 vaccination prior to infection in protecting against adverse perinatal outcomes, and the number of pregnant individuals in our cohort. To carry out the calculation, we needed to supply a value for the effect size that was independent of our cohort. According to a systematic review and meta-analysis, rates of adverse perinatal outcomes were 15% higher in unvaccinated pregnant individuals than among those who were vaccinated^46^. Therefore, our post-hoc power analysis assumed that the rate of such outcomes would be at least 15% higher among pregnant women who were not vaccinated. The null hypothesis was that the rates of adverse outcomes would be the same among vaccinated and unvaccinated pregnant people. In the present cohort of 227 pregnancies, approximately one-third of the participants were vaccinated before COVID-19 infection.

We carried out a post-hoc power analysis using G*Power 3.1.9.4^47^. Our calculations assumed a 5% false-positive rate and used a two-tailed test of the difference in proportions between the vaccinated and unvaccinated groups. The results indicated that our analysis had 90% power, in other words, the chance of rejecting the null hypothesis if it was false was 90%. This rate is reasonably high and we believe it provides support for our conclusions about this cohort.

Data was analyzed using R language^48^. Statistical analysis was conducted using a combination of epiDisplay package^49^, tableone package^49^, stats base package, and aod package^50^.

### Proteomic profiling analysis

The present data is reanalyzed from a dataset previously published by our group^7^. We conducted a proteomic reanalysis to explore potential associations between respiratory distress and canonical pathways possibly associated with SARS CoV-2 in a subset of infants for whom proteomics was performed. We analyzed a subset of 45 SEU infants born in the first year of the pandemic (April 2020 to March 2021) matched to seven control infants born to unexposed healthy women at the pandemic onset, for a total of 52 infants (Fig. 1). Controls were from a convenience sample of healthy mothers who did not have SARS-CoV-2 exposure and for whom infant specimens were available. Infants were matched based on gestational age. This analysis utilizes peripheral infant blood specimens collected between 24 and 48 h of life. This timeframe was selected to coincide with routine bilirubin checks in order to minimize blood draws. The SEU infant cohort was clustered according to RD outcome and gestational age, resulting in four groups: no RD term infants (*n* = 27), no RD preterm infants (*n* = 3), RD term infants (*n* = 4), and RD preterm infants (*n* = 11). Significant differentially expressed proteins between healthy and the four COVID-19-exposed infant groups were determined by two-tailed Mann Whitney *U* test using the R base package, t.test, considering fold-change ≥2 and FDR-adjusted *p* value < 0.05. Enrichment analysis was conducted using the online platform Enricher^51^. Comparisons between multiple groups using 1-way ANOVA with uncorrected Fisher’s test in GraphPad Prism v9.4.0. Network and pathways analyses were performed using QIAGEN Ingenuity Pathway Analysis (IPA) v01-19-00^7,42^.

### Ethics and inclusion statement

This research was conducted locally in Los Angeles, California and was approved by the UCLA Internal Review Board. The authors come from diverse socioeconomic backgrounds and have expertise in a variety of disciplines across medicine and public health. This manuscript cites prior studies from across the United States, including Los Angeles, with an emphasis on sources about maternal and child health. Our results are locally relevant with the goal of providing physicians and patients more information about the benefits of COVID-19 vaccination. Furthermore, it can help guide future research and understandings of the long-term sequelae of SARS-CoV-2.

The roles and responsibilities of the authors were agreed upon prior to conducting the analysis. This project and future projects have been designed with special attention to continue to train medical students, medical residents and fellows, graduate students, and post doctorial researchers.

The data collection process and analysis methods did not result in increased stigmatization, incrimination, discrimination, or other personal risks to our study participants. This study did not cause increased health, safety, or security risks to the researchers or participants. No animal welfare, environmental protection, or biorisk-related regulations were violated by our study. Our study did not involve the transfer of biological materials, cultural artefacts, or associated traditional knowledge.

### Reporting summary

Further information on research design is available in the Nature Portfolio Reporting Summary linked to this article.

### Supplementary information

Supplementary Information

Peer Review File

Reporting Summary

## Data Availability

The data that support the findings of this study are available under restricted access due to the sensitivity of information and patient confidentiality. The raw data are protected and not available due to data privacy laws. Access to processed deidentified data may be available upon reasonable request to the corresponding authors. The raw data used for the proteomic profiling analysis were published previously^7^ and are publicly available (10.17632/mdnb359tp9.1). Data are located in a controlled access electronic storage managed by the University of California Los Angeles Health System.

## Code availability

The code for data analysis associated with the manuscript may be available upon reasonable request to the corresponding authors.
