# Supplementary material for: Respiratory distress in SARS-CoV-2 exposed uninfected neonates followed in the COVID Outcomes in Mother-Infant Pairs (COMP) Study
Source: Nat Commun. 2024 Jan 24;15:399. doi: 10.1038/s41467-023-44549-5 (PMC10808093; doi:10.1038/s41467-023-44549-5)
Supplement: Supplementary file 1 — Supplementary Information [file 41467_2023_44549_MOESM1_ESM.pdf]

SUPPLEMENTAL TABLES AND FIGURES:

**SUPPLEMENTARY TABLE 1:** Maternal and infant demographics stratified by maternal vaccination status			
Variables	Unvaccinated n=118	Vaccinated n=68	P value*
Infant Outcomes			
Male Sex (%)	60 (50.8)	31 (45.6)	0.590
Vaginal Delivery (%)	71 (60.2)	43 (63.2)	0.797
Preterm Delivery < 37 wks	26 (22.0)	7 (10.3)	0.069
Low birth weight (<2500 g)	27 (22.9)	4 (5.9)	0.005
Maternal Predictors			
Age (mean yrs (s.d.))	32.32 (6.4)	33.79 (5.0)	0.105
Ethnicity			0.507
Asian, mixed race, or other	25 (21.4)	17 (25.0)	
Black, Hispanic, or Latino	62 (53.0)	30 (44.1)	
White	30 (25.6)	21 (30.9)	
Pulmonary Hypertension	0 (0.0)	1 (1.5)	0.780
Autoimmune disorder	11 (9.3)	6 (8.8)	1.000
HIV positive	2 (1.7)	0 (0.0)	0.733
Obesity pre pregnancy	41 (34.7)	8 (11.8)	0.001
Diabetes	5 (4.2)	3 (3.3)	1.000
Congenital Heart Disease	6 (5.1)	2 (2.9)	0.750
Asthma	13 (11.0)	10 (14.7)	0.614
Substance Use	3 (2.5)	5 (7.6)	0.219
Pregnancy Complications			
Chronic or Gestational Hypertension	38 (32.2)	21 (30.9)	0.982
Preeclampsia or HELLP	22 (18.6)	8 (11.8)	0.307
Gestational Diabetes	16 (13.6)	14 (20.6)	0.295
Chorioamnionitis	9 (7.6)	5 (7.4)	1.000
Postpartum Hemorrhage	14 (11.9)	3 (4.4)	0.151
COVID Predictors			
Vaccination prior to delivery	7 (6.0)	68 (100.0)	<0.001
Trimester of SARS-CoV-2 infection			0.180
First or second	62 (52.5)	28 (0.41)	
Third	56 (47.5)	40 (58.8)	
COVID severity			0.131
Asymptomatic, Mild, or Moderate			
Severe or Critical	12 (10.2)	2 (2.9)	
COVID symptoms			
Fever	33 (28.7)	17 (27.9)	1.000
Cough, Rhinorrhea, Congestion	81 (71.1)	56 (91.8)	0.003

Dyspnea	29 (25.0)	6 (9.8)	0.027
Nausea/Vomiting, Abdominal Pain, Diarrhea	20 (17.5)	5 (8.2)	0.145
Anosmia or Dysgeusia	33 (28.9)	7 (11.5)	0.015
Treatment			
Remdesivir	12 (10.2)	5 (7.4)	0.706
Dexamethasone	10 (8.5)	1 (1.5)	0.104
Convalescent Plasma	3 (2.5)	0 (0.0)	0.471
Monoclonal Antibodies	6 (5.1)	8 (11.8)	0.169
Paxlovid	1 (0.8)	2 (2.9)	0.626
Variant			<0.001
Ancestral and Others	93 (80.2)	2 (3.0)	
Alpha	1 (0.9)	0 (0.0)	
Delta	11 (9.5)	15 (22.4)	
Omicron	11 (9.5)	50 (74.6)	

*p-values were calculated based on one-sided Fisher's exact test. We did not adjust for multiple comparisons because it was exploratory.

SUPPLEMENTARY FIGURE 1: Distribution of infants with and without respiratory distress across gestational ages by hypertensive disorders of pregnancy (n=199).

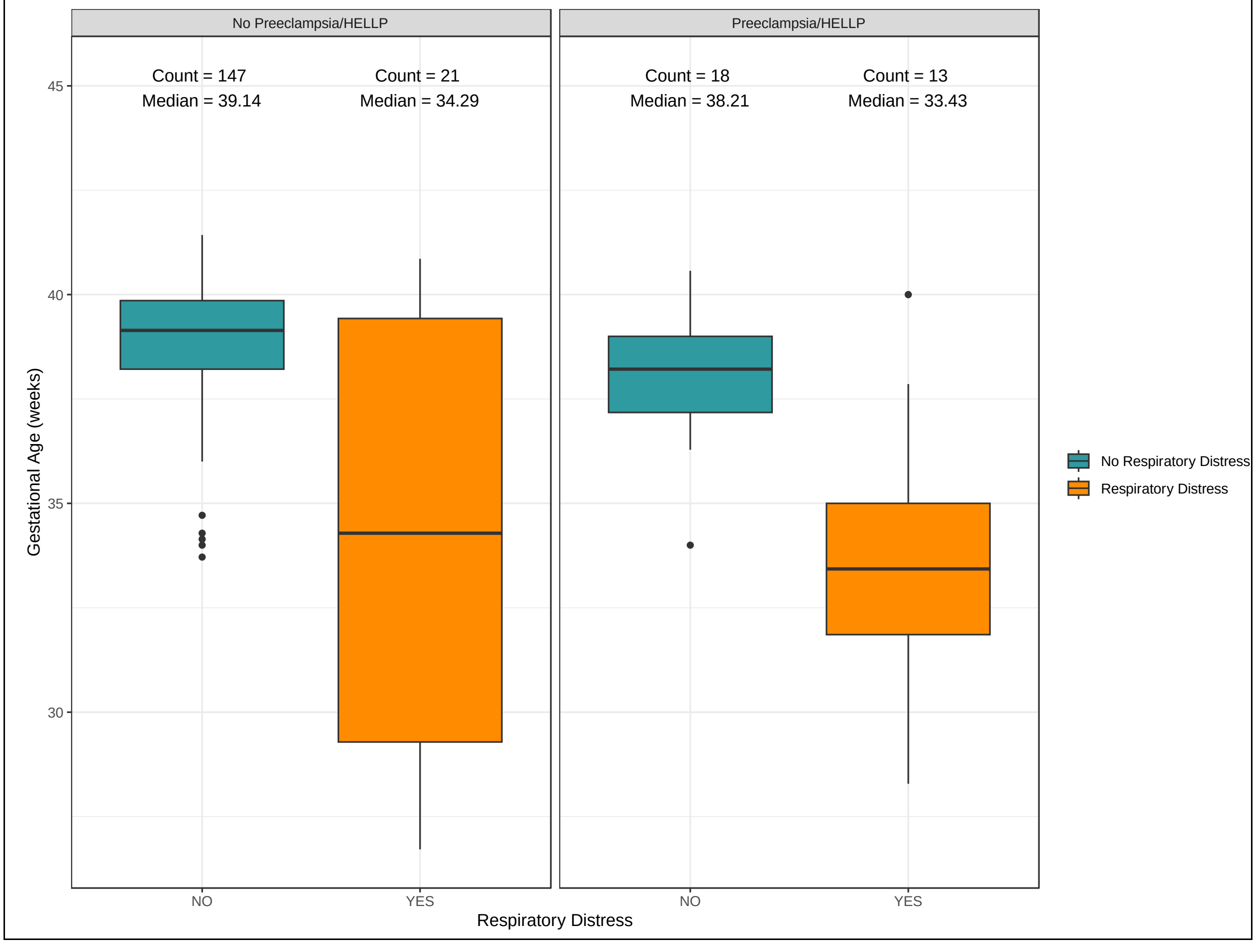

SUPPLEMENTARY FIGURE 2: Hypothesized relationship between maternal SARS-CoV-2, hypertensive disorders of pregnancy, prematurity, and neonatal respiratory distress

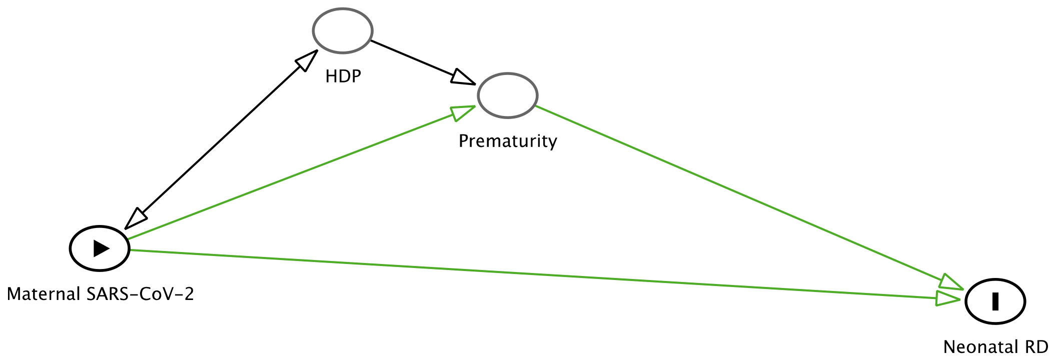

HDP: hypertensive disorder of pregnancy; RD: respiratory distress

Our hypothesized relationship between these variables is threefold: 1. There is a large overlap between the clinical presentations of HDP and severe SARS-CoV-2, which can make it difficult to differentiate between these entities clinically; 2. It is both possible that patient's with HDP may have more severe manifestations of COVID and that COVID may exacerbate HDP; 3. Both HDP and COVID can increase the risk for prematurity.

Created with daggity.com

SUPPLEMENTARY FIGURE 3: Univariate logistic regression analysis of respiratory distress in neonates born to women infected with COVID during pregnancy (n=199).

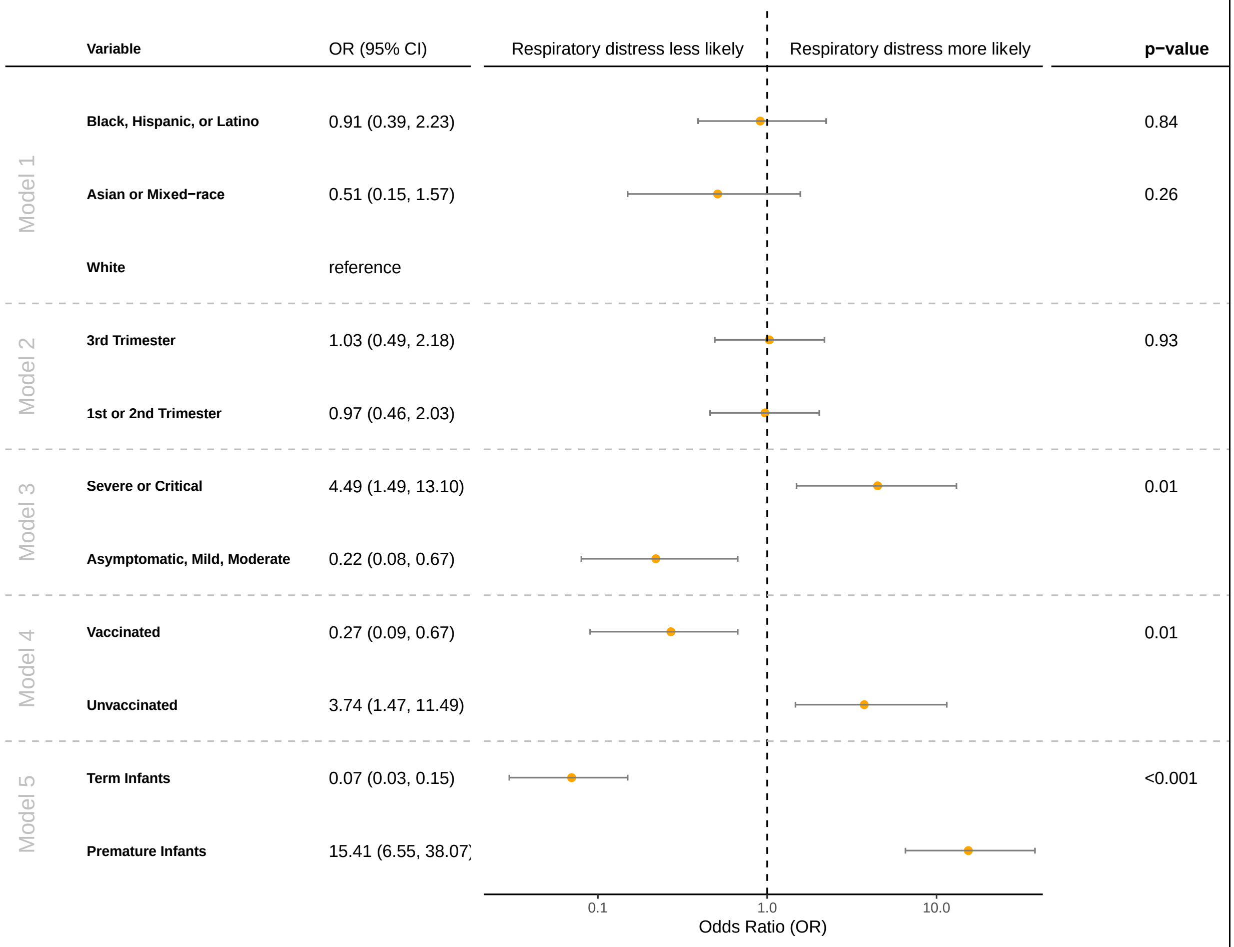

OR: odds ratio; CI: Confidence interval

Data is presented as the OR +/- 95% CI; p-values are calculated based on a one sided Wald's test.

COVID severity index was determined based on the NIH classification system[41], as done by Thompson et al.[4] and described in our methods. Briefly, critical illness describes patients with respiratory failure or signs of multiple organ failure, severe illness was defined as patients with desaturations requiring supplemental oxygen, moderate illness included patients with dyspnea or who were admitted to an inpatient unit for COVID, mild illness describes symptomatic patients not meeting criteria for more severe disease, and asymptomatic individuals showed no symptoms.

SUPPLEMENTARY FIGURE 4: Multivariable logistic regression analysis of respiratory distress in neonates born to women infected with COVID during pregnancy (n=199).

Variable	Adjusted OR (95% CI)	p−value
**Severe/Critical**	2.10 (0.55, 7.62)	1.00
**Preterm Infants**	13.73 (5.71, 34.56)	<0.001

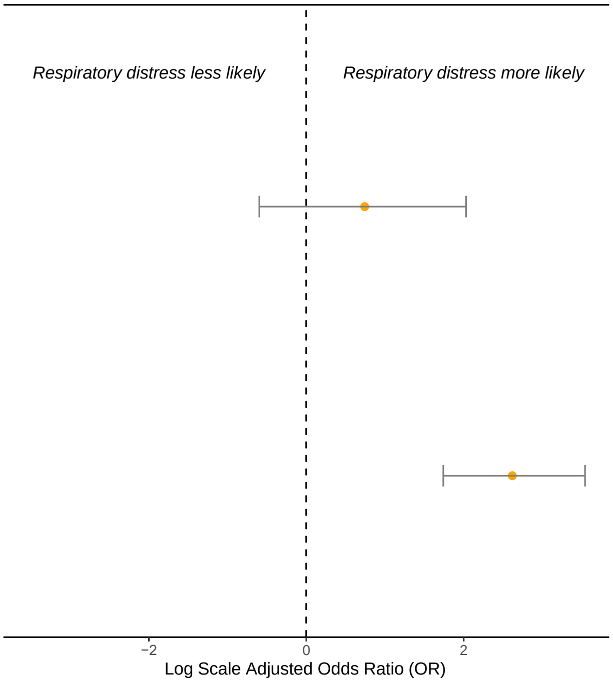

OR: odds ratio; CI: Confidence interval

Data is presented as the OR +/- 95% CI; p-values are calculated based on one sided Wald's test

Multivariable logistic regression model included maternal COVID-19 severity and infant prematurity as predictors for respiratory distress.

Severe/Critical refers to mothers who had signs of respiratory failure, multiorgan failure, or desaturations requiring supplemental oxygen compared to mothers who had moderate/mild/or asymptomatic disease (reference group).

Preterm infants refers to neonates born at less than 37 weeks gestation compared to term infants (reference group) born at greater than or equal to 37 weeks gestation.
