# Supplementary material for: Respiratory distress in SARS-CoV-2 exposed uninfected neonates followed in the COVID Outcomes in Mother-Infant Pairs (COMP) Study
Source: Nat Commun. 2024 Jan 24;15:399. doi: 10.1038/s41467-023-44549-5 (PMC10808093; doi:10.1038/s41467-023-44549-5)
Supplement: Supplementary file 3 — Reporting Summary [file 41467_2023_44549_MOESM3_ESM.pdf]

nature portfolio

Corresponding author(s): Olivia M Man
Mary Catherine Cambou

Last updated by author(s): Nov 30, 2023

Reporting Summary

Nature Portfolio wishes to improve the reproducibility of the work that we publish. This form provides structure for consistency and transparency in reporting. For further information on Nature Portfolio policies, see our Editorial Policies and the Editorial Policy Checklist.

Statistics

For all statistical analyses, confirm that the following items are present in the figure legend, table legend, main text, or Methods section.

n/a	Confirmed	
☐	☒	The exact sample size (*n*) for each experimental group/condition, given as a discrete number and unit of measurement
☐	☒	A statement on whether measurements were taken from distinct samples or whether the same sample was measured repeatedly
☐	☒	The statistical test(s) used AND whether they are one- or two-sided<br>*Only common tests should be described solely by name; describe more complex techniques in the Methods section.*
☐	☒	A description of all covariates tested
☐	☒	A description of any assumptions or corrections, such as tests of normality and adjustment for multiple comparisons
☐	☒	A full description of the statistical parameters including central tendency (e.g. means) or other basic estimates (e.g. regression coefficient) AND variation (e.g. standard deviation) or associated estimates of uncertainty (e.g. confidence intervals)
☐	☒	For null hypothesis testing, the test statistic (e.g. F, t, r) with confidence intervals, effect sizes, degrees of freedom and P value noted<br>*Give P values as exact values whenever suitable.*
☒	☐	For Bayesian analysis, information on the choice of priors and Markov chain Monte Carlo settings
☒	☐	For hierarchical and complex designs, identification of the appropriate level for tests and full reporting of outcomes
☒	☐	Estimates of effect sizes (e.g. Cohen's d, Pearson's r), indicating how they were calculated

Our web collection on statistics for biologists contains articles on many of the points above.

Software and code

Policy information about availability of computer code

Data collection	Information was obtained directly from participants using RedCap survey software and from clinical chart review using EPIC.
Data analysis	Statistical data was analyzed in Data was analyzed using R language. Statistical analysis was conducted using a combination of epiDisplay package, tableone package, stats base package, and aod package. Proteomic assessment was conducted using an online platform Enricher, GraphPad Prism, and QIAGEN Ingenuity Pathway Analysis

For manuscripts utilizing custom algorithms or software that are central to the research but not yet described in published literature, software must be made available to editors and reviewers. We strongly encourage code deposition in a community repository (e.g. GitHub). See the Nature Portfolio guidelines for submitting code & software for further information.

Data

Policy information about availability of data

All manuscripts must include a data availability statement. This statement should provide the following information, where applicable:

- Accession codes, unique identifiers, or web links for publicly available datasets
- A description of any restrictions on data availability
- For clinical datasets or third party data, please ensure that the statement adheres to our policy

The data that support the findings of this study are available under restricted access due to sensitivity of information and patient confidentiality. The raw data are protected and not available due to data privacy laws. Access to processed deidentified data may be available upon reasonable request to the corresponding

authors. The raw data used for the proteomic profiling analysis were published previously and are publicly available (https://dx.doi.org/10.17632/mdnb359tp9.1).

Data is located in a controlled access electronic storage managed by the University of California Los Angeles Health System.

Research involving human participants, their data, or biological material

Policy information about studies with human participants or human data. See also policy information about sex, gender (identity/presentation), and sexual orientation and race, ethnicity and racism.

Reporting on sex and gender	Our cohort comprised of pregnant persons and their neonates. None of the mothers in our study self-identified as non-binary or trangender; therefore, we have used gendered language in our text to refer to the mothers.
Reporting on race, ethnicity, or other socially relevant groupings	Maternal race and ethnicity was operationalized into three categories ("Black, Hispanic, and Latina", "Asian, Mixed-Race, and Other", or "White") based on self-reported racial identity. We acknowledge that race is a social construct and our categorizations may not adequately reflect an individual's identity. However, we included race in our univariate analysis given the history of systemic racism that has contributed to poor maternal outcomes among black women in the United States.
Population characteristics	Pregnant individuals, greater than or equal to 16 years old or older, with confirmed SARS-CoV-2 infection during gestation were eligible for enrollment, regardless of preexisting conditions. Participants were primarily recruited by the Department of Obstetrics at the University of California, Los Angeles (UCLA) from 15 April 2020 to 31 August 2022. We did not exclude participants based on preexisting co-morbidities. Two-hundred and twenty-one pregnant individuals, aged 16 to 56 years old, and 227 SARS-CoV-2 exposed fetuses were enrolled in our study. This resulted in 199 live births following in utero exposure to COVID-19.
Recruitment	Participants were primarily recruited by the Department of Obstetrics at the University of California, Los Angeles (UCLA) from 15 April 2020 to 31 August 2022. Beginning in April 2020, all women admitted to UCLA labor and delivery were screened for SARS-CoV-2 by nasopharyngeal swab. Beginning in April 2020, all women admitted to UCLA labor and delivery were screened for SARS-CoV-2 by nasopharyngeal swab. The UCLA Medical Center comprises of multiple teaching hospitals, including tertiary and quaternary referral centers, and services the Los Angeles region in Southern California.<br><br>The majority of pregnant individuals in our cohort were enrolled from a tertiary and quaternary medical center in Los Angeles, California. Several mother-infant pairs were transferred from community hospitals in the county due to severity of their symptoms. Therefore, our study population may be skewed towards more severe presentations of COVID-19 compared to the general pregnant population. On the other hand, due to the high level of specialized services and medical equipment available, our study may also have higher rates of survival when compared to other regions.
Ethics oversight	Informed consent for participation was obtained for all participants prior to enrollment. If a participant was incapable to provide consent (i.e., due to an acute hospitalization or intubated), consent was provided by a surrogate decision maker and the participant was re-consented once they regained capacity. Information was obtained directly from participants using RedCap survey software and from clinical chart review using EPIC.

Note that full information on the approval of the study protocol must also be provided in the manuscript.

Field-specific reporting

Please select the one below that is the best fit for your research. If you are not sure, read the appropriate sections before making your selection.

☒ Life sciences ☐ Behavioural & social sciences ☐ Ecological, evolutionary & environmental sciences

For a reference copy of the document with all sections, see nature.com/documents/nr-reporting-summary-flat.pdf

Life sciences study design

All studies must disclose on these points even when the disclosure is negative.

Sample size	We enrolled all mothers infected with SARS-CoV-2 during pregnancy and their infants who presented to the UCLA Health system.
Data exclusions	We did not exclude mothers or their children from this study. Occasionally, participants were lost to follow-up if they decided to receive medical care elsewhere.
Replication	This was an observational study. The data processing code (created in R language) has been rerun multiple times and to yield our findings.
Randomization	We compared the demographics of infants born with and without RD using one way t-tests. We considered variables related to infant characteristics (sex, delivery method, prematurity, low birth weight), maternal predictors (maternal age, ethnicity, preexisting medical conditions), pregnancy complications (e.g., preeclampsia, gestational hypertension, chorioamnionitis, etc.), and COVID predictors (maternal vaccination, trimester of infection, severity, symptoms, treatment, viral variant). Next, we conducted a logistic regression multivariable analysis. We selected variables to include in our final model using a backwards selection and WALDs test. We prioritized variables that were significant from the prior t-tests and based on clinical suspicion of intermediate variables and effect modifiers. We considered potential collinearity or lack of independence among predictor variables using chi-squared tests for independence
Blinding	This was an observational study, participants were not blinded into groups.

Reporting for specific materials, systems and methods

We require information from authors about some types of materials, experimental systems and methods used in many studies. Here, indicate whether each material, system or method listed is relevant to your study. If you are not sure if a list item applies to your research, read the appropriate section before selecting a response.

Materials & experimental systems

n/a	Involved in the study
☒	☐ Antibodies
☒	☐ Eukaryotic cell lines
☒	☐ Palaeontology and archaeology
☒	☐ Animals and other organisms
☒	☐ Clinical data
☒	☐ Dual use research of concern
☒	☐ Plants

Methods

n/a	Involved in the study
☒	☐ ChIP-seq
☒	☐ Flow cytometry
☒	☐ MRI-based neuroimaging
